# Impact of horticultural therapy on patients admitted to psychiatric wards, a randomised, controlled and open trial

**DOI:** 10.1038/s41598-024-65168-0

**Published:** 2024-06-22

**Authors:** Aude Joubert, Blandine Jankowski-Cherrier, Audrey Rossi, Laure Teyssier, Valérie Suraud, Emilie Presle, Romain Pommier, Catherine Massoubre, Elise Verot

**Affiliations:** 1 CHU Saint-Etienne, Pôle de Psychiatrie, Psychiatrie Secteur plaine – CMP, CATTP Et Hôpital de Jour Andrézieux, 42055 Saint-Etienne, France; 2 CHU Saint-Etienne, Pôle de Psychiatrie, Unité d’admission N°3 - Secteur Plaine, 42055 Saint-Etienne, France; 3 Department of Clinical Investigation Centre, CIC 1408-INSERM Clinical Investigation Centre, CIC 1408-INSERM, University Hospital of Saint-Etienne, Saint-Etienne, France; 4 REHALise, Centre Référent de Réhabilitation Psychosociale de Saint-Etienne, Rehacoor42, CHU de Saint-Etienne, Saint-Etienne, France; 5 Laboratoire TAPE-EA 7423, Université Jean Monnet, Service Universitaire CHU Saint-Etienne, Saint-Etienne, France; 6 Laboratoire Parcours Santé Systémique, Université Claude Bernard Lyon 1, Université de Lyon, P2S UR 4129, 69008 Lyon, France; 7 Chaire Hygée, Institut PRESAGE, Université Jean Monnet, Université de Lyon, 42023 Saint-Etienne, France; 8 Laboratoire Parcours Santé Systémique, Université Jean Monnet, Université de Lyon, P2S UR 4129, 42270 Saint-Etienne, France; 9 Unité de Recherche Clinique, Pharmacologie Clinique, Centre Hospitalo-Universitaire de Saint-Etienne, Saint-Etienne, France; 10 Nursing Sciences and Health Technics Department, Faculty of Medicine, Jean Monnet University, 42271 Saint Priest en Jarez, France

**Keywords:** Horticultural therapy, Inpatients, Nursing, Psychiatry, Psychological distress, Psychiatric disorders, Anxiety, Therapeutics, Rehabilitation

## Abstract

Psychiatric inpatients often endure anxiety. This randomized trial assessed the impact of horticultural therapy on anxiety in adult psychiatric inpatients over four weeks, compared to standard care. Recruiting 211 inpatients from six units were randomized into control (n = 105) and experimental (n = 106) groups. Control received usual care; the experimental group had horticultural therapy alongside usual care. Anxiety, measured using HADS-A scale at four weeks, aimed to establish horticultural therapy's superiority. After four weeks, horticultural therapy significantly reduced anxiety compared to standard care (*P* < 0.001). These results argue in favor of integrating horticultural therapy into psychiatric nursing practices.

*Trial registration*: No Clinical Trail: NCT02666339 (1st registration: 28/01/2016).

## Introduction

Some patients suffering from a psychiatric disorder require hospitalization. In these moments of "decompensation," stress, anguish and anxiety are exacerbated^1^. Irrespective of psychiatric pathology, hospital care contributes to increased levels of stress and anxiety in the hospitalized patient^1^. This is due to confinement in new places, forced hospitalizations, compulsory treatment, and the inability to be able to act, to control one's life^1^. Anxiety manifests itself differently in various individuals and is frequently found in patients hospitalized in psychiatric wards^2^.

Inpatient psychiatric treatment and counselling are used as the first line of treatment, but other non-medication therapies can also be suggested, such as horticultural therapy^3^. According to the American Horticultural Therapy Association, horticultural therapy is defined as a person's engagement in gardening and plant-based activities, facilitated by a qualified therapist, to achieve specific therapeutic goals^3^. Various theories, inspired by work on the original Man/Plant co-relation, the vital cooperation process of Phyto-resonance or Biophilia, explain how horticultural therapy acts on stress levels, anguish and anxiety^4^. All these theories agree that "relationships with nature are a fundamental component of building or maintaining 'good health'"^4^. Immersion in nature brings into play a polysensory solicitation that favors the emotional and affective openness of the individual, through emotional processes allowing access to a feeling of security and stress reduction^5,6^. Activities linked to plant cultivation have proved to have beneficial therapeutic effects for many people, with a wide range of profiles, both in terms of age and pathologies^7^. In their review of the literature, Annerstedt and Währborg^8^ point to many disparities in the way horticultural therapy is implemented. They find differences in terms of the activities proposed, the practices mobilized, the assessment tools and the population studied.

Sempik has shown that horticultural therapy can promote physical and psychological well-being^9^. However, controlled studies have seldom been conducted. The literature review by Annerstedt and Währborg^8^ highlighted a significant therapeutic effect of horticultural therapy on well-being, self-esteem, quality of life, emotional stability, and reduction in symptoms of depression and anxiety. These authors point out that there is also an impact on psychiatric pathology ^8^. In chronic pain patients, Verra et al.^10^ assessed the effect of horticultural therapy on anxiety, using the Hospital Anxiety and Depression Scale (HADS)^11,12^. Indeed, the intergroup effect size for the HADS-A (the specific part of the HADS for measuring anxiety) was measured at 0.23 (ES = 0.26 in the horticultural therapy group versus 0.03 in the control group; p = 0.043) after four weeks of treatment. Kam and Siu found that 10 sessions of horticultural therapy over a two-week period reduced anxiety, stress and depression in patients with psychiatric disorders, the majority of whom suffered from schizophrenia^13^. However, the population studied was not hospitalized and continued to work^13^. We therefore hypothesized that patients receiving horticultural therapy sessions in addition to their usual care during their psychiatric hospitalization would result in a significant reduction in their anxiety, regardless of their psychiatric pathology (psychotic disorder, mood disorder, personality disorder, anxiety disorder).

The aim of our study is to evaluate the effect of mediation by horticultural therapy on the anxiety state of patients hospitalized in adult psychiatry, compared with the effect of usual care (current practice) after four weeks of mediation or usual care.

## Methods

### Design

The Melisses Garden study was a prospective, monocentric, controlled, randomized, open-label trial in two parallel groups, carried out in six psychiatric units for adults in the same hospital. The two parallel groups of the study included one group receiving horticultural therapy and a control group receiving usual care.

### Participants

Patients were recruited from an adult psychiatric unit comprising six hospital wards in Saint-Etienne (France).

Patients were eligible for inclusion if they met the following criteria: (1) minimum age of 18 years old, (2) a planned hospital stay of at least one month, (3) a HADS-A anxiety score greater than 8 (cut off point), (4) medical condition sufficient to enable beginning mediation, (5) ability to give informed consent to participate in the study or, for patients under guardianship, obtainment of informed consent from the guardian, (6) affiliation with or entitlement to membership of a social security program, (7) up-to-date tetanus vaccination (verified by a rapid test carried out prior to randomization).

The exclusion criteria for patients were (1) refusal of the randomization procedure, (2) inability to complete the scales for whatever reason (non-French speaking, mental retardation, etc.), (3) behavior assessed by psychiatrist did not allow the indication of mediation, (4) previous participation in horticultural therapy mediation.

### Ethical statement

The study was performed in accordance with the Declaration of Helsinki, and its protocol was approved under the reference No. EUDRACT: 2016-A00057-44. Written informed consent was obtained from the patients before starting the trial. This study was registered on Clinical Trial Registry code ClinicalTrials.gov ID: NCT02666339, 28/01/2016.

### Measures

#### Hospital anxiety and depression scale (HADS)

The HADS is a self-administered test including 14 items in total. The HADS consists of two subscales, the HADS-A (Anxiety subscale) and the HADS-D (Depression subscale)^12^. Seven items assess anxiety, of which two assess autonomic anxiety (panic and butterflies in the stomach), and the remaining five assess tension and restlessness^14^. Scores for items in each subscale of the HADS are calculated to produce an anxiety score (HADS-A) or a depression score (HADS-D); the two measurements can be also be added together to produce a total score (HADS-T).

This user-friendly, accessible scale is sensitive to measuring change, and can therefore be used to monitor shifts in anxiety and/or depression symptomatology over time or during treatment^15^. Studies have demonstrated the effectiveness of this scale in the general population^12^ as well as in those suffering from various pathologies^12,16–18^. Bedford et al.^19^ reported on values found in patients with psychiatric disorders, and identified distinct values in each part of the scale. The mean HADS-A score was 13.9, with a standard deviation of 4.4. This scale also proved its test–retest reliability (r > 0.80), even at two weeks^20^. A psychometrically validated French version of the scale exists^21^. For Barczack et al.^22^, a score of 8 or more represents the optimum threshold, with sensitivities of 82% and 70% and specificities of 94% and 68% for anxiety and depression, respectively.

This study shows the discriminating effect of the scale between anxiety and depression. Bedford et al.^19^ observed a mean HADS-A of 13.9 ± 4.4 in psychiatric inpatients. To our knowledge, the HADS, with a specific focus on subscale A, has not been used in the psychiatric adult population. This validated, easy-to-use scale seems to be the most relevant for measuring the anxiety state of patients suffering from psychiatric disorders by nurses and other health care staff members in the context of this study.

### Sample size

To enable us to show a moderate effect of horticultural therapy, meaning a difference of 2 points on the HADS-A scale (Cohen's effect size d = 0.5)^23^ in this population, it was necessary to include at least 84 patients per group, or 168 in total, for an alpha risk of 5% and a power of 90%. In total, considering an attrition rate of approximately 10%, 190 patients were to be included (95 per group).

### Recruitment and randomization

The referral nurses of the Melisses garden group, present in each psychiatric unit, assessed the suitability of each hospitalized patient's participation in horticultural therapy mediation. They then shared an indication completed by the patient's referring psychiatrist with the multidisciplinary team, authorizing the study to be suggested to the patients. The patient was then given the study's information leaflet, and a period of at least 48 h in which to reflect on his or her decision. Consent was signed once the Clinical Research Assistant had ensured that the patient had fully understood the research protocol. Once the patient had signed the protocol, the observation notebook was opened and all documents anonymized (identified by monogram and inclusion number). Patients were then asked to complete the HADS-A scale to assess their anxiety score. Six researchers were responsible for patient recruitment and the random allocation of patients to either the intervention or control group.

**Figure 1:**
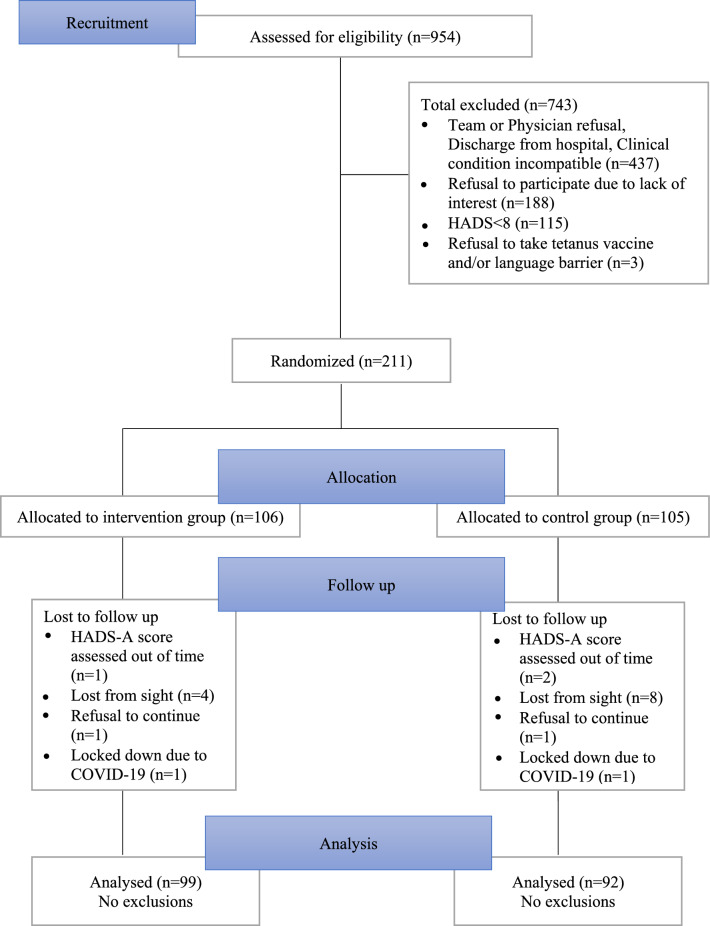
Flow diagram for participants in the study.

The randomization procedure was computer-generated using SAS software (SAS-Windows® version 9.4 on PC). Graphs were produced using R® software version 4.0.2. The randomization list, implemented in REDCap software, consisted of a pseudorandom computer list drawn up before the start of the study. Randomization was balanced by blocks of variable size and was centralized via an e-CRF. The CONSORT (Consolidated Standards of Reporting Trials) flow diagram^24^ was followed to manage the random allocation (Fig. 1).

### Procedures

#### Intervention group

Patients in the intervention group received horticultural therapy along with routine nursing care, the details of which are illustrated in Table 1 below.

**Table 1:** Overview of the intervention divided on control group and horticultural therapy group.

	Day 0	Week 1	Week 2	Week 3	Week 4	Day 0 + 1 month
Control group	HADS-A > 8					HADS-A
	Inclusion					
Horticultural therapy group	HADS-A > 8					HADS-A

Routine nursing care

Horticultural therapy

In addition to the horticultural therapy sessions, the patient received the same treatment as the control group. They benefited from therapeutic mediations other than horticultural therapy, which had been decided upon prior to randomization. Horticultural therapy sessions were offered at the rate of two one-and-a-half-hour sessions per week for four weeks. The number of patients managed was six per session, supervised by two nurses. Each session was structured along the same lines: welcome (arrival of patients), reminder of what had been achieved and observed during the previous session, warm-up (performing a few movements related to gardening gestures), mediation with a theme (weeding, planting, mulching, cuttings, watering, ikebana, etc.), clean-up and tidying up of tools, followed by an assessment of the session (tasks performed, patients' feelings, schedule for the next session).

#### Control group

Patients assigned to the control group benefited from standard practices: medical interviews, nursing interviews, drug therapy and therapeutic mediations (art therapy, music therapy, cooking, access to social space, concomitant day hospital, medical-psychological center, adapted physical activity). Any alternative therapeutic mediation (other than horticultural therapy) deemed necessary for the patient was decided upon before randomization.

#### Data collection and analyses

Study data were collected and managed using REDCap electronic data capture tools hosted at university hospital of Saint-Etienne^25,26^. REDCap (Research Electronic Data Capture) is a secure, web-based software platform designed to support data capture for research studies, providing (1) an intuitive interface for validated data capture; (2) audit trails for tracking data manipulation and export procedures; (3) automated export procedures for seamless data downloads to common statistical packages; and (4) procedures for data integration and interoperability with external sources.

Data were collected between September 2016 and June 2021. The trial was designed to establish superiority of horticultural therapy over standard of care to reduce the anxious state of the patient hospitalized in adult psychiatry, measured by the HADS-A scale at four weeks.

All analyses were performed using the complete case population (all randomized patients with available data).

The HADS-A scale at four weeks (primary outcome) is a quantitative outcome and was compared between the two groups with the use of the Student’s t-test. The difference of means and its 95% confidence interval were estimated. In addition, an analysis of covariance was also performed to adjust the result obtained for the primary outcome on the baseline HADS-A scale and the main psychiatric pathologies. A multiple imputation of missing data was performed, in a sensitivity analysis, for the analysis of the primary outcome for the intention-to-treat population analysis. All analyses were 2-sided and a P value of less than 0.05 was considered statistically significant. Analyses of secondary outcomes were reported as unadjusted mean difference and 95% CIs, without P values. Differences should be considered exploratory and not clinically directive. Statistical analyses were performed using SAS software, version 9.4 software (SAS Institute Inc).

### Ethics approval and consent to participate

The study was performed in accordance with the Declaration of Helsinki, and its protocol was approved under the reference No. EUDRACT: 2016-A00057-44. Written informed consent was obtained from the patients before starting the trial. This study was registered on Clinical Trial Registry code ClinicalTrials.gov ID: NCT02666339.

## Results

Of the 954 patients assessed for eligibility, 634 were offered the protocol. 423 patients were not included due to a refusal to participate, a HADS score < 8, hospital discharge or worsening clinical condition. 211 patients were randomized to the study: 106 to the horticultural therapy group and 105 to the control group.

### Participants’ characteristics

The median age was 42.9 years old, and the male–female distribution was equitable. The median time from hospitalization to entry into the study was 17 days. Most of the patients had previous gardening experience: 67.0% and 76.2%, in the horticultural therapy group and in the control group, respectively. At the time of inclusion, the characteristics of the two groups were well-balanced, except for professional activity and main place of residence. Thirty-three patients (31.1%) had a professional activity in horticultural therapy group and 50 patients (48.1%) in the control group. In the horticultural therapy group, 42 participants (40.0%) lived at home, compared with 26 patients (24.8%) in the control group. In contrast, 45 patients (42.9%) in the control group lived in an apartment with a balcony or terrace.

An overview of the baseline demographic and clinical characteristics of patients is presented in Table 2.

**Table 2:** Baseline demographic and clinical characteristics of patients.

Characteristics	Horticultural therapy (N = 106)	Standard of care (N = 105)
Median age (IQR^a^)—years	41.1 (31.1–51.3)	44.4 (35.3–53.9)
Male—No. (%)	53/105 (50.5)	52/105 (49.5)
Principal place of residence—No. (%)		
House	42/105 (40.0)	26/105 (24.8)
Apartment with balcony	29/105 (27.6)	45/105 (42.9)
Apartment without balcony	33/105 (31.4)	33/105 (31.4)
Other	1/105 (1.0)	1/105 (1.0)
Professional activity—no. (%)	33/106 (31.1)	50/104 (48.1)
Previous experience in gardening—No. (%)	71/106 (67.0)	80/105 (76.2)
Pathology that motivated the admission to psychiatry—No. (%)		
Depression	42/102 (41.2)	45/100 (45.0)
Personality disorders	16/101 (15.8)	16/102 (15.7)
Anxiety disorders	9/101 (8.9)	12/100 (12.0)
Schizophrenia	9/100 (9.0)	7/100 (7.0)
Other	30/101 (29.7)	28/103 (27.2)
Median time from randomization to psychiatric admission (IQR^a^)—days	16.0 (9.0–30.0)	18.0 (10.0–37.0)

^a^IQR = Interquartile Range.

### Effect of the horticultural therapy on patient’s anxiety at 4 weeks (primary outcome)

At 4 weeks, mean HADS-A was 9.55 ± 4.10 in the horticultural therapy group (n = 99 data available) and 11.02 ± 4.39 in the control group (n = 92 data available, difference statistically significant (mean difference = (−1.48, 95% CI −2.68 to −0.27, *P* = 0.017). This difference remained statistically significant with an adjusted analysis on the initial HADS-A (*P* < 0.001) and with an adjusted analysis on the initial HADS-A and the types of pathology (*P* = 0.005).

In a sensitivity analysis, given that 9.5% (20/211) of data on primary outcome were missing and in order to observe the result on the intention-to-treat population, a multiple imputation of missing data was performed. Thus, the consistency of the result was verified, with a statistically significant result (*P* = 0.017; *P* < 0.001 and *P* = 0.001 for respectively the unadjusted analysis, adjusted on the initial HADS-A and adjusted on the initial HADS-A and the type of pathology). To tack into account the imbalance observed at inclusion, the analysis adjusted for these imbalanced factors was performed and showed a result statistically significant (*P* = 0.012). In total, 36 patients (36.4%) had a HADS-A score ≥ 11 at 4 weeks in the horticultural therapy group and 51 (55.4%) patients in the control group.

Effects of the horticultural therapy on patients’ anxiety are illustrated in Table 3 and detailed scores for each group are illustrated in Table 4.

**Table 3:** Outcome assessment of anxiety and length of hospital stay: case-complete analysis.

Variables and time points	Horticultural therapy (N = 106)	Standard of care (N = 105)	Mean difference between groups (95% CI^a^)	*P* value
Anxiety (HADS-A^a^)				
At inclusion—mean (SD^a^)	13.7 (2.9)	12.7 (2.9)		
Primary outcome at 4 weeks^b^—mean (SD^a^)	9.55 (4.10)	11.02 (4.39)	−1.48 (−2.68 to −0.27)	0·017
Secondary outcome at 8 weeks^c^—mean (SD^a^)	9.56 (4.64)	10.59 (3.91)	−1.03 (−2.33 to 0.26)	
Length of hospital stay in days				
Mean (SD^a^)	47.3 (24.4)	48.3 (27.6)	−1.00 (−9.03; 7.02)	
Median (IQR^a^)	43.0 (32.0–58.0)	42.5 (29.0–56.0)		

^a^SD = Standard Deviation. IQR = Interquartile Range. CI = confidence interval. HADS-A = Hospital Anxiety and Depression Scale—Anxiety.

^b^Datas were available for 99 patients in the horticultural therapy group and 92 in the standard of care group.

^c^Datas were available for 86 patients in the horticultural therapy group and 81 in the standard of care group.

**Table 4:** Details of general anxiety score on the HADS-A scale.

	Horticultural therapy (N = 106)	Standard of care (N = 105)	Odds ratio (95% CI)
HADS-A inclusion—no. (%)			
0 to 7	0	0	0
8 to 10	17/106 (16)	30/105 (28.6)	47/211 (22.3)
11 or more	89/106 (84)	75/105 (71.4)	164/211 (77.7)
HADS-A at four weeks—no. (%)			
0 to 7	33/99 (33.3)	23/92 (25.0)	1.00
8 to 10	30/99 (30.3)	18/92 (19.6)	1.16 (0.53 ; 2.56)
11 or more	36/99 (36.4)	51/92 (55.4)	0.49 (0.25; 0.97)

### Secondary outcomes

At 8 weeks, mean HADS-A was 9.56 ± 4.64 in the horticultural therapy group and 10.59 ± 3.91 in the control group (mean difference = (−1.03, 95% CI −2.33 to −0.26). The median length of hospital stay was 43.0 days in the horticultural therapy group and 42.5 days in the control group.

## Discussion

To our knowledge, our study is the first randomized, controlled clinical trial to investigate the impact of horticultural therapy on the anxiety state of adult psychiatric inpatients. Although numerous randomized and non-randomized studies have already been carried out, mostly in Asia and Oceania, they have involved few patients and focused on depression, mainly in the elderly demographic^27^. However, they were able to demonstrate that horticultural therapy had a significant positive effect on the outcome of depressive symptoms in the elderly. Our results confirm the positive effect of horticultural therapy on mental health, given that both anxiety and depression are pathologies affecting the individual's mental health. Furthermore, our results show that horticultural therapy has a significant effect on reducing anxiety in adult psychiatric inpatients, regardless of the psychiatric pathology they suffer from. At four weeks, 36.4% of patients in the horticultural therapy group had a HADS score above 11, compared with 55.4% in the control group, while the percentages of scores above 11 at inclusion were 84% and 71.4. Several studies have shown that horticultural therapy significantly reduces anxiety in students^28^, middle-aged women^29^ and the general population^30^. Our results therefore added to what is already known on the subject. Other psychiatric studies have also demonstrated the effectiveness of horticultural therapy in combination with drug treatments^31^. We can draw a parallel with our results, which show that the reduction in anxiety is enhanced by horticultural therapy, regardless of whether medication is considered.

A recent meta-analysis^32^ concluded that horticultural therapy has a positive effect on mental health. This meta-analysis collated 18 Randomized Controlled Trials including heterogeneous populations (ages, pathologies) and different assessment tools (biological markers, muscle strength, etc.), located outside the field of psychiatry^32^. The specificity of our study focuses on the anxiety of adult psychiatric inpatients, which to our knowledge has never been previously investigated.

Studies have furthermore demonstrated the impact of horticultural therapy on reducing high blood pressure, heart rate and salivary cortisol levels in children and elderly people with mental health problems^33–36^. The impact of horticultural therapy on sleep^34^, depression^27^, mood enhancement^37^, improving physical capabilities^35^, stress^29,30,35^ and improving quality of life^30^ has already been evaluated. The results are significant, confirming that therapeutic gardening and gardening activities have a positive impact on overall human health.

Our study reinforces the field of knowledge on the subject by providing evidence of a significantly positive impact of horticultural therapy on the anxiety of adults hospitalized in psychiatric wards. Given the fact that the activity can be easily replicated and is inexpensive, we suggest that policymakers invest in this prevention strategy aimed at maintaining the overall health of psychiatric service users and the general population.

Finally, if we refer to Leclerc's article^38^, the higher the level of anxiety, the more likely it is that the sequence of events will lead to an act of violence. The correlation of these data leads us to hypothesize that horticultural therapy in care settings can reduce violence by acting on anxiety levels. Nursing staff, and specifically nurses, therefore, have a role to play in therapeutic patient education to maintain a low to moderate level of anxiety. Horticultural therapy as a non-medication intervention has a scientific value that can be widely disseminated. This lowering of anxiety levels could lead to a reduction in hetero- and auto-aggressive behavior, and would therefore be a means of combating the isolation and restraint of hospitalized patients in psychiatry^39^.

### Limitations

The present study has its limitations. The calculation of our sample size did not take into account that so many patients would leave the study prematurely, and one of the difficulties encountered was the reduction in the average length of hospital stays in line with the development of outpatient care between 2016 and 2021. In fact, over the six years of research, the average length of stay fell from 41 to 29 at our inclusion center, resulting in a loss of 10% of our patients.

Our trial did not take into account biological measures such as biomarkers, cortisol measurement and blood pressure measurement, which may prove to be a limitation. However, as mentioned above, other studies have shown convincing results of horticultural therapy on biological markers^33–36^ and we have chosen to focus on anxiety to contribute to our knowledge base.

Despite these limitations, this study enabled all participating psychiatric patients of all pathologies to benefit from horticultural therapy, allowing us to generalize the results of the study to all people with moderate to severe mental illness. The study population was sufficiently large to have achieved significant results. However, it seems necessary to consider this study as a preliminary evaluation of horticultural interventions as a complement to standard psychiatric care.

Although the results are promising, a major limitation of this study is its preliminary nature. As such, it serves primarily to explore the potential benefits of horticultural interventions rather than to provide definitive evidence of their effectiveness. The results obtained cannot be generalised without further validation on a larger scale. A major challenge lies in the difficulty of distinguishing the specific effect of horticultural therapy from the therapeutic mediation activities offered in the control group. In this study, standard care often included group mediation activities on the theme of art, which may also have positive effects on patients' anxiety. Thus, it is possible that the effects observed are partly attributable to general therapeutic interventions rather than to horticultural therapy per se. To overcome this limitation, it would be necessary to design comparative studies in which the control group performs no such therapeutic activities, or to introduce more specific, non-therapeutic control interventions.

## Conclusion

Patients hospitalized in psychiatry are subject to known anxiety, generated by various factors such as the experience of hospitalization, the constraint of hospitalization, the institutional setting, medication (non-exhaustive list). This study provides evidence of the efficacy of horticultural therapy on the anxiety level of patients hospitalized in psychiatry for a minimum of four weeks. Additional research is warranted to explore the impact of horticultural therapy on the consumption of anxiolytic treatments, which could also influence the reduction in the duration of hospitalization.

## Data Availability

The data that support the findings of this study are available from the corresponding author upon reasonable request.
